# ODMSummary: A Tool for Automatic Structured Comparison of Multiple Medical Forms Based on Semantic Annotation with the Unified Medical Language System

**DOI:** 10.1371/journal.pone.0164569

**Published:** 2016-10-13

**Authors:** Michael Storck, Rainer Krumm, Martin Dugas

**Affiliations:** Institute of Medical Informatics, University of Münster, Münster, Germany; Flinders University, AUSTRALIA

## Abstract

**Introduction:**

Medical documentation is applied in various settings including patient care and clinical research. Since procedures of medical documentation are heterogeneous and developed further, secondary use of medical data is complicated. Development of medical forms, merging of data from different sources and meta-analyses of different data sets are currently a predominantly manual process and therefore difficult and cumbersome. Available applications to automate these processes are limited. In particular, tools to compare multiple documentation forms are missing. The objective of this work is to design, implement and evaluate the new system ODMSummary for comparison of multiple forms with a high number of semantically annotated data elements and a high level of usability.

**Methods:**

System requirements are the capability to summarize and compare a set of forms, enable to estimate the documentation effort, track changes in different versions of forms and find comparable items in different forms. Forms are provided in Operational Data Model format with semantic annotations from the Unified Medical Language System. 12 medical experts were invited to participate in a 3-phase evaluation of the tool regarding usability.

**Results:**

ODMSummary (available at https://odmtoolbox.uni-muenster.de/summary/summary.html) provides a structured overview of multiple forms and their documentation fields. This comparison enables medical experts to assess multiple forms or whole datasets for secondary use. System usability was optimized based on expert feedback.

**Discussion:**

The evaluation demonstrates that feedback from domain experts is needed to identify usability issues. In conclusion, this work shows that automatic comparison of multiple forms is feasible and the results are usable for medical experts.

## Introduction

Medical documentation is a complex and heterogeneous field [1,2] applied in different settings such as patient care using electronic health records (EHR) or clinical trials employing electronic data capture (EDC) systems. Furthermore, it is the basis for quality management and certification purposes in the clinical setting.

Data collected in patient care is applied for different purposes including clinical research [3]. Documentation procedures evolve over time and are subject to modifications. The knowledge gained from clinical research should be considered in the development of new data items in EHR systems. Design of forms, merging of data from different sources and meta-analyses of different data sets is currently a predominantly manual process and therefore difficult and cumbersome. To foster reuse of medical forms, a meta-data repository for medical forms called Medical Data Models portal (MDM portal, http://medical-data-models.org) was introduced. Currently it contains over 6700 medical forms [4–6]. Given this high number of medical forms and items, manual comparisons of items for a set of forms are practically not possible.

Electronic analysis of medical forms can be applied to identify data items with potential for secondary use, i.e. the application of routine clinical data for research purposes. Recently, an automated Unified Medical Language System (UMLS) based application for comparison of medical forms called compareODM was implemented, which is able to analyze relations between items of two forms [7,8]. This tool is based on the Operational Data Model standard (ODM) from the Clinical Data Interchange Standards Consortium (CDISC) [9] and has been applied to study documentation patterns for prostate and breast cancer [10]. In this tool, items were annotated with medical concepts to analyze not only name or value domain but also the similarity regarding the concept domain. However, compareODM is restricted to compare two medical forms at a time, provides limited output capabilities and was not evaluated regarding usability. A new and extended system has to be designed and implemented to enable comparison of multiple forms and increase the usability and availability of such a tool to a greater audience.

### Objectives

So far, tools for automatic analysis of medial forms are limited to the comparison of two forms, although the application in clinical registries or multi-center studies would require the comparison of multiple medical forms. Given the high number of data elements in medical forms that need to be compared, a clear presentation of similarities between medical forms has to be developed. Because the analysis of medical forms requires extended knowledge on the medical domain, the software has to be evaluated by medical experts. In summary, the objective of this work is to design, implement and evaluate a system, called ODMSummary, for comparison of multiple forms with a high number of data elements, which can be applied in the context of data exchange and has a high level of usability for medical experts.

## Materials and Methods

### Requirements for automatic item comparison

Comparison of data elements has to be performed by medical experts with domain knowledge to interpret the corresponding results.

The following requirements were identified by reviewing the features of compareODM and conducting non-structured expert interviews on multiple form comparison. The system must be capable to summarize a set of forms, to enable the user to track changes in different versions of forms and to find comparable items in different form sets. Output shall be clear and easily interpretable. Installation and configuration efforts shall be as small as possible. To achieve a clear and understandable presentation of results, unnecessary and redundant information shall be removed. In summary, the goal of this software is to provide a tool, which supports physicians and researchers in the following use cases:

- Overview of the documentation content and estimation of documentation effort for multiple forms,
- Comparison of different versions of a form set,
- Comparison of form sets between different documentation tasks (e.g. routine documentation vs. trial documentation),
- Comparison of form sets between different institutions, and
- Identification of comparable items between different form sets (e.g. secondary use, data exchange and transformation).

### Representation of forms in ODM format

Comparison of multiple medical forms is implemented based on the ODM format [9] which is used by various EDC systems. The Define-XML standard, an extension of the ODM standard, is accepted by the Food and Drug Administration as a metadata standard for clinical data study definition [11] and implements study designs in a standardized, machine-readable manner. Currently, this standard is evaluated for electronic exchange of trial data [12]. Potentially, thousands of medical research studies with hundreds of thousands of medical forms and corresponding items are available in ODM standard format. The required semantic annotation of items is based on concept codes from the UMLS, which includes around 3.25 million medical concepts [13].

### Concept domain and value domain

**Fig 1:**
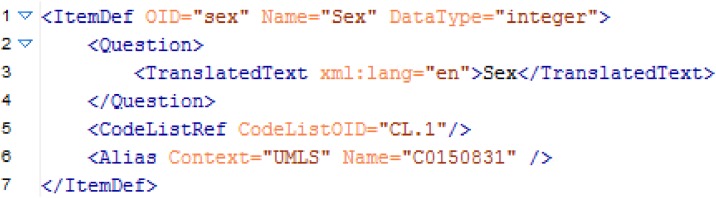
An exemplary item definition for the item sex in ODM format.

ODM is an XML format and a medical item, for instance “Sex”, may look like the code snippet in Fig 1. In this example the following information is relevant to compare items:

1. Name of item (Name = "Sex"),
2. Data type of item (DataType = "integer"), and
3. Concept of item (Alias Context = "UMLS" Name = "C0150831").

The concept of this exemplary item is marked as an UMLS code (Context = "UMLS") and the code of this item is “C0150831”. According to the ISO/IEC 11179 standard one item can be represented by multiple codes and this set of UMLS codes is called the concept domain of the item. The value domain of the item is characterized by the data type and the optional code list [14–16].

**Fig 2:**
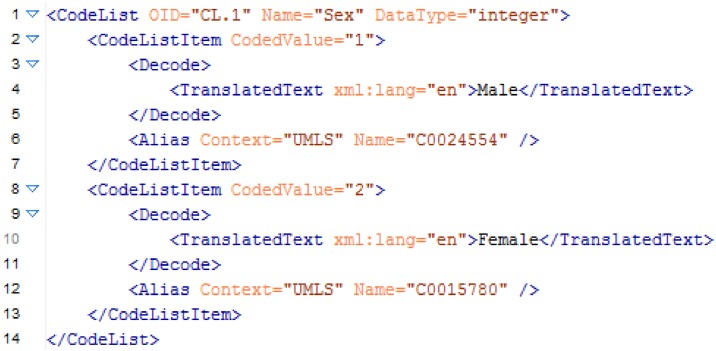
An exemplary code list definition for the item sex in ODM format.

The referenced code list of the example item (Sex) is shown in Fig 2. In this example the following information is relevant to compare code lists:

1. Name of the code list (Name = "Sex"),
2. Data type of the code list (DataType = "integer"), and
3. Different code list items, which are represented by
  - a coded value and
  - a set of UMLS codes.

To make it easy to decide for the user if data elements are comparable or not, we implemented categories for different levels of similarity. The lowest similarity level for two data elements is called **SIMILAR** implying identical concept domains for both data elements. Since **MATCHING** and **TRANSFORMABLE** data elements share the value domain, data stored for data elements with this similarity level can be merged. The highest similarity level is **IDENTICAL** which includes not only same concept and value domain but also the same item name. Summarizing item comparison can be categorized into the following item to item relationships (detailed explanation in S1 File):

- **IDENTICAL** (Data stored for the two items is identical.)
- **MATCHING** (Data stored for the two items can be merged without transformation.)
- **TRANSFORMABLE** (Data stored for the two items can be combined by data transformation.)
- **SIMILAR** (The concept domain of the two items is identical.)
- **DIFFERENT** (The concept domain of the two items is different.)
- **NOTCODED** (The item is not coded, so it cannot be compared.)

### Algorithm

The compare algorithm passes through all given ODM files and compares every specified item with all other items in these ODM files. Item relationship is determined for every combination of items. Thus, an overview is generated how each item compares to every other item in the given set of ODM files.

**Fig 3:**
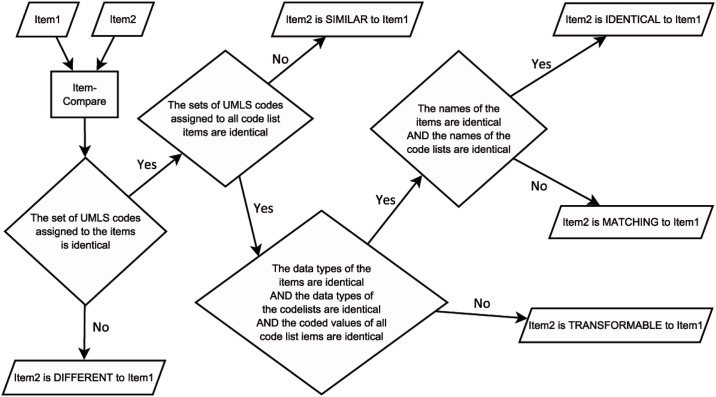
Flowchart of the ODMSummary compare algorithm.

Initially, all items are checked for semantic annotation. Items without semantic codes are marked as **NOTCODED** and excluded from the comparison algorithm. The flowchart in Fig 3 presents an overview of the implemented compare algorithm. Comparison of items is done pairwise, so the algorithm requires two items in every step of comparison. As a first step, the semantic codes of two items are compared. If the coding differs, these items are set as **DIFFERENT**, because they describe different medical concepts. In a second step all semantic codes attached to the code lists of the two given items are checked. Items are declared to be **SIMILAR** if the medical concepts are identical, but the codes within the attached code lists or the data type mismatch. Otherwise, if codes within the attached code lists fit, items are called **TRANSFORMABLE** because stored data for these items can be transformed back and forth using data transformation. Items are called **MATCHING** if—in addition to identical semantic coding of their code lists—data types of these items and their code lists and all coded values of corresponding code list items are the same. Data of matching items can be combined without a transformation. Finally, items are **IDENTICAL** when also the names of these items and their code lists are the same (case-insensitively).

### Evaluation of usability

ODMSummary was developed to create an user-friendly and easy-to-use software for a wide group of users. 12 medical experts were invited to participate in the evaluation of the software tool regarding usability. For this purpose, medical experts had to solve given tasks with ODMSummary (tasks in S2 File) including the analyses of different versions of one given medical form and the identification of reusable items in forms of various documentation fields and institutions. The evaluation of ODMSummary was conducted in three phases: In phase 1, medical experts completed the given tasks applying ODMSummary embedded in the MDM portal. Since major difficulties occurred in completing the tasks during evaluation phase 1, those medical experts received the output of the comparison from phase 1 tasks as an Excel file (Tasks in S3 File) in a second phase of evaluation. These were the same tasks as in phase 1 but leaving out the first part of the tasks requiring generating the results. In each phase, medical experts were asked to comment on difficulties occurring during evaluation. In phase 3, a workshop was run with 6 of the 12 medical experts to discuss and solve usability issues. Afterwards a final evaluation was conducted with the tasks from phase 1 by 12 additional medical experts, which were not involved in the previous implementation and discussion process. To measure usability of the system, participants were asked to fill out System Usability Scale (SUS) questionnaires [17]. This questionnaire consists of ten questions and provides a result value between 0 and 100, whereby 0 is worst and 100 is the best imaginable usability.

## Results

**Fig 4:**
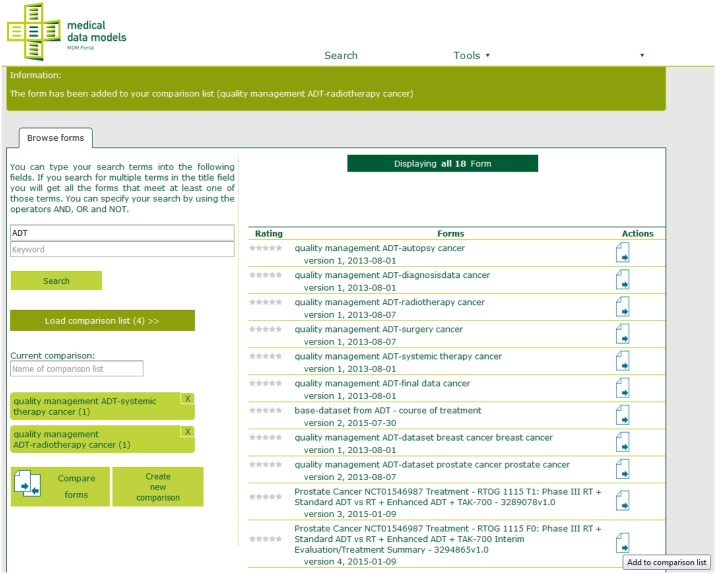
Screenshot from the Medical Data Model (MDM) Portal with embedded ODMSummary functionality. A form comparison list is presented on the left side. Forms can be added easily to this comparison list (Actions buttons on the right side).

ODMSummary was developed as a tool for summarizing and comparing ODM files in form of a web application written in JAVA, which is easily accessible over the internet. The comparison can be called directly from the website or as a web service which returns the result of the comparison to the calling system. As an example, it can be directly called from the MDM portal [4–6]. ODMSummary enables the comparison of all semantically annotated forms within the MDM portal. The integration of the tool is shown in Fig 4. Forms can be easily added to a comparison list and saved.

### Summary of forms’ content

**Fig 5:**
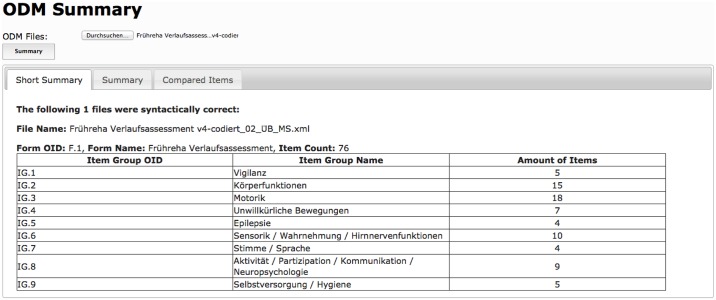
Short summary of a forms’ content. Item groups and amount of items are presented for each input file.

In the first place the algorithm of ODMSummary validates ODM input files against the XML standard and the XML schema definition (XSD) of ODM standard (Supported versions: 1.3.1 [18] and 1.3.2 [19]). If at least one file is syntactically incorrect, the system returns validation error messages for the corresponding file. A detailed report about analyzed forms is provided for standard compliant input files. The report is split in two tabs called “Short Summary” and “Summary”. An overview of item groups including the amount of items is displayed within “Short Summary” (see Fig 5).

**Fig 6:**
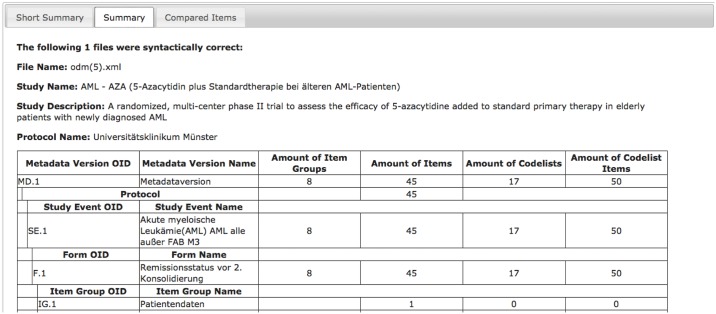
Summary of ODM-files’ structure.

In addition, the summary page presents information about the study, study events and metadata versions and how the different ODM structures like item groups, items and code lists are referenced to each other (Fig 6). This function allows determining the workload for documentation with a certain form, to check if a form covers a certain topic and if documentation of this topic is feasible with this form. For example, to prepare a clinical trial the documentation forms can be evaluated regarding completeness and documentation effort.

In summary, ODMSummary allows to determine the amount and complexity of items within forms, provides an overview on documentation content and enables to assess the documentation effort for multiple forms.

### Data element comparison for multiple forms

**Fig 7:**
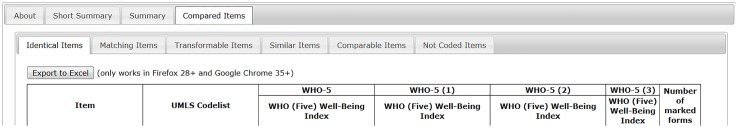
Result of multiple forms comparison.

ODMSummary is a tool for comparison of multiple forms at the same time with any number of items. Given the large number of items in medical documentation, a clear summary and presentation of comparison results is essential. As shown in Fig 7 results are grouped by item-to-item relationship (“identical”, “matching”, etc.) and a summary of all relationships is given in an additional tab called “Comparable Items”. The user can decide which relationships are relevant for his/her use case and focus on these results.

**Fig 8:**
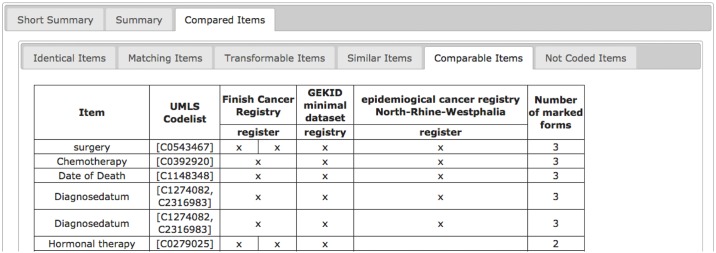
Output of a form comparison showing comparable items in three different registries. The three registries include several comparable items like “Chemotherapy” and the Finish Cancer Registry even contains the item “surgery” twice.

In Fig 8 exemplary output for comparable items is presented. The table provides information on study name, study type, compared items and the corresponding UMLS codes. The first column specifies compared items with a corresponding list of UMLS codes in column two. Headings in column 3 to 5 refer to the study names and form names (e.g. register). Each row represents the comparison result for a single item. An “x” marks each form which includes an item comparable to the compared item. Using on-mouse—over, a tooltip is shown with detailed information on the compared item (1^st^ column) or the item of the examined form.

In case of more than one form per study, the corresponding column is split into the number of available forms. The column is split as well if one form includes more than one comparable item, as shown for item "surgery" in the Finish Cancer Registry (Fig 8). Thus, displaying similarity levels for all combinations of data elements from multiple forms is achieved.

### Use cases

In the following paragraphs, functions and feasibility of ODMSummary are explained with the mentioned use cases.

#### Comparison of different versions of a form set

As documentation evolves over time it is useful to track changes between different versions. Particularly, identifying items that switch from one form to another is important. With ODMSummary it is possible to analyze the content of documentation sets and its development.

**Fig 9:**
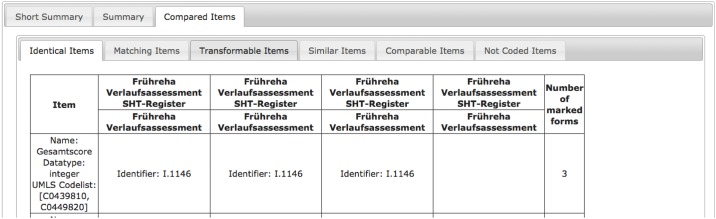
Comparison of 4 versions of a form regarding item “Gesamtscore” (eng. total score). It is available in identical manner in versions 1,2 and 3.

**Fig 10:**
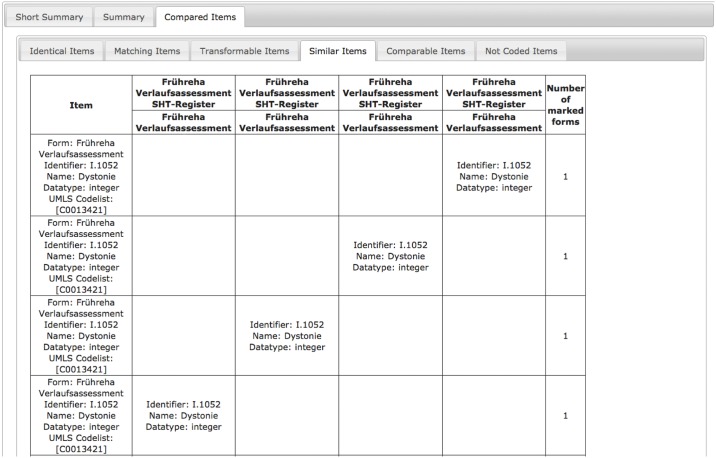
Comparison of 4 versions of a form showing the development of the item “Dystonie” (eng. dystonia).

An example is displayed in Fig 9. The identical item “Gesamtscore” (eng. total score) is included in the first 3 versions of the form and missing in its latest. If the content of an item changes, it can be tracked as well. In Fig 10, this has been analyzed for the item “Dystonie” (eng. dystonia). This item has different code lists in different versions of the form, i.e. same concept domain, but different value domain.

#### Comparison of form sets between different documentation types (e.g. routine documentation vs. trial documentation)

**Fig 11:**
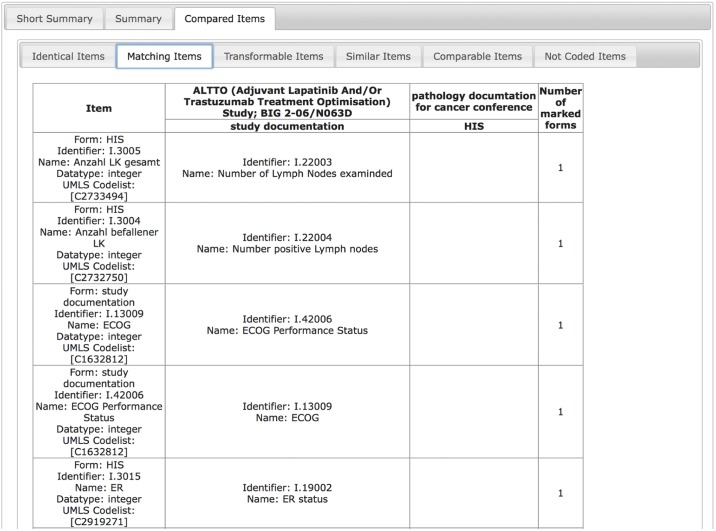
Concepts for „Number of Lymph Nodes examined“, „Number of positive Lymph nodes“, as well as ECOG Status and ER status are matching between clinical pathology documentation and trial documentation.

ODMSummary can be applied to identify comparable items within different documentation types. In Fig 11, a comparison of clinical (pathology report) with clinical trial documentation is presented. This analysis allows gaining insight whether the necessary documentation for a clinical trial can be covered by clinical routine data and if this data can be applied for secondary use.

In this example, the „Number of Lymph Nodes examined”and the „Number of positive Lymph nodes”are matching between clinical pathology and clinical trial documentation.

#### Comparison of form sets between different institutions and identification of comparable items between different form sets (e.g. secondary use, data exchange and transformation)

**Fig 12:**
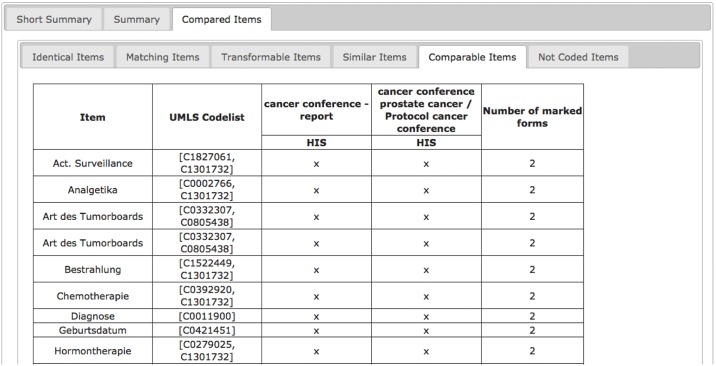
Comparable Items of forms for the same domain (cancer) from different institutions. The comparable items are “Act. Surveillance”, “Analgesics”, “Type of the Tumour Board”, “Irradiation”, “Chemotherapy”, Diagnosis”, Birthdate” and “Hormone therapy”.

ODMSummary can be used to analyze forms from different institutions. In particular, this is helpful to detect differences and similarities between documentation in multicenter studies. The result of this comparison can help to improve collaboration and exchange of data between these cooperating institutions. Fig 12 presents an example for this use case and refers to two protocols applied in cancer documentation in two university hospitals. A variety of data items is comparable in these two forms, so the data could be exchanged and reused in multicenter studies.

Form comparison is also applicable within the documentation landscape of a clinic or for a specific disease. Established documentation procedures can be adapted to prevent duplicate collection of the same data in one clinic. Another advantage is the detection of comparable items for secondary use. Therefore, this tool can contribute to avoid additional workload by redundant and extensive documentation for quality management, registries or clinical trials.

#### Implementation of a user-friendly software and evaluation of usability

ODMSummary has been developed with a focus on user-friendliness. In the process of evaluation, 12 medical experts were invited to participate. In the first phase of evaluation we asked these medical experts to solve 4 given tasks with ODMSummary (Tasks in S2 File). Each of these tasks includes a question about a comparison of different form sets. Tasks were accompanied by a step by step instruction how to perform the comparison with ODMSummary and fulfill the tasks.

Although the medical experts got instructions, these 12 experts reported difficulties to complete the tasks. Only one participant has solved the tasks correctly. The first difficulties appeared performing the registration process for the MDM portal and using the file upload form for multiple files. The most relevant problem was the interpretation of the comparison’s output. Due to the large amount of data presented it was difficult for the participants to find the appropriate information.

In the second round an Excel export function was implemented to adapt the tasks given in the first round, i.e. the output of ODMSummary was presented as an Excel sheet (Tasks in S3 File). Those medical experts received the results of the ODM forms’ comparison from tasks 1 and were asked for interpretation. During this phase of evaluation, major usability issues occurred again. One drawback of the approach is that useful information like tooltips and additional information given on the website are not available in the Excel sheet which could have contributed to the difficulties.

After two rounds of testing a workshop with medical experts was conducted to discuss and solve usability issues. This discussion lead to a restructuring of the result pages: main information (item’s name) is always visible to the user and additional information like the data type is hidden in tooltips. So the page is not overloaded with information and can be interpreted more easily. Furthermore, an explanation page with information about purpose and functionality of the system and an example output has been developed.

After redesigning the output and establishing an explanation page a last evaluation was conducted with 12 additional medical experts re-using the tasks from phase 1. All 12 participants were able to solve the four tasks correctly and filled out the System Usability Scale questionnaire. The results of the questionnaire are shown in Table 1 varying between 40 and 97.5 points. The mean SUS score for all participants is 74.2 which is considered a good usability score. [17]

**Table 1:** Results of the System Usability scale in the last evaluation phase.

SUS_1	SUS_2	SUS_3	SUS_4	SUS_5	SUS_6	SUS_7	SUS_8	SUS_9	SUS_10	SUS_Score
4	2	4	1	5	1	5	1	4	2	87.5
3	1	5	1	4	1	4	1	3	2	82.5
4	1	5	1	5	1	5	1	5	1	97.5
5	3	4	2	4	1	4	2	4	1	80
3	3	4	2	3	2	4	2	3	2	65
2	1	5	1	4	2	5	2	3	1	80
4	2	5	2	4	1	5	1	5	1	90
2	3	3	3	2	4	3	4	4	4	40
2	3	4	2	3	3	2	3	1	3	45
4	1	4	3	5	1	4	2	3	1	80
3	2	5	2	4	2	4	1	3	2	75
2	2	4	1	2	2	3	2	4	1	67.5

## Discussion

Comparison of medical forms is important for their evaluation and improvement. Development of medical forms should be based on existing forms and items to use experiences from previous projects, to save time and to facilitate reuse of medical data. Therefore, systematic comparison is needed to analyze existing forms, to find reusable items and to facilitate reuse of metadata and thereby contribute to compatible data collection. These analytic tools should not only be available to technical staff but especially to medical experts, who have the knowledge to interpret and enhance medical items and forms. Existing medical forms should be disclosed and made available as open source to foster reuse of metadata in all types and fields of documentation. This would save a lot of time invested in the development of new medical forms and enhance existing documentation [20].

ODMSummary is based on the previous published compareODM algorithm, but has been completely new developed, since one major limitation of compareODM is its’ availability in only one offline software environment—R (R Package compareODM, http://cran.r-project.org). Thus, the application of compareODM requires knowledge in R programming [8]. The new implementation provides a website to upload ODM files and retrieve the results of comparison directly. Furthermore the tool can be called as a service, for example as an implementation in the MDM portal [4–6].

A novel functionality of ODMSummary is simultaneous comparisons for any number of forms and items. In particular, multiple forms specified in one ODM-file can be handled by ODMSummary, while compareODM only takes the first form of an ODM-file into account. Moreover, the check for similarity between items is more precise due to the implementation of a new similarity level called transformable. This is beneficial for statistical analysis, because data of transformable items can be joined easily.

Another approach to compare information from heterogeneous forms is the application of ontologies like Basic Formal Ontology (BFO) [21], Suggested Upper Merged Ontology (SUMO) [22] or Descriptive Ontology for Linguistic and Cognitive Engineering (DOLCE) [23]. Following the definition of Brookes and Robinson UMLS metathesaurus also is an ontology because it combines conceptualization of domain entities with interrelationships among those entities [7,24]. But, ODMSummary takes the relationships between concepts not into account because a relationship between concepts is not sufficient to find data elements for secondary use, in particular for automated data exchange and transformation. For example, the concepts “headache” and “urticaria” are both children of the concept “symptom”. The distance of these two terms in the conceptual graph is relatively small; however, these are two different data elements concerning reuse of data. Lexical databases like WordNet [25] enable measuring the distance between two terms. However, lexical-based approaches have major limitations for item comparison of medical forms because of homonyms (e.g. "size" can be body height, tumor size of foot size) and abbreviations (e.g. laboratory values). In contrast, semantic codes from UMLS can provide clarity for item comparison. UMLS includes common medical classifications and nomenclatures like SNOMED CT [26] or LOINC [27], which leads to a good coverage of specific medical terms. However, in UMLS not all medical terms are available, so they cannot be coded with the proposed approach. To deal with this coding issue, ODMSummary shows data elements without semantic annotations to support manual review of these data elements.

ODMSummary has been developed to improve medical documentation. It is now possible to get a quick overview over a set of forms and their documentation fields due to the structured overview of forms and items given by ODMSummary. Advanced comparison of multiple forms enables medical experts to compare forms or whole datasets for reuse and scientific purposes. The evaluation has shown that the development of systems used by domain experts can only be successful if the experts are involved in the development process. The adjustments to solve usability issues were developed closely with domain experts to fulfill the needs of the designated user group. Thus, we have shown that an automatic form comparison of multiple forms is feasible and the results can be presented understandable for medical experts.

### Limitations and future work

The presented approach has limitations that should be mentioned: The comparison method is limited to forms in ODM format. However, the ODM standard is common for the representation of case report forms [28] and the Define-XML standard—an extension of the ODM standard—is accepted by the Food and Drug Administration as a metadata standard for clinical data study definition [11]. Furthermore, all items within these forms need to be semantically annotated with the same coding approach. Another potential drawback of this work is that the form’s structure, e.g. position of the data element in the form, was not considered for the comparison of data elements. But, the structural information should be part of the semantic annotations if it is relevant for the meaning of the specific data element. UMLS is not a classification, so it includes synonyms, which leads probably to different semantic coding for items of the same meaning. Tools for uniform semantic annotation [29] can be applied to mitigate this issue. Nevertheless, it is possible to expand the implementation to apply other semantic coding strategies with different terminologies like SNOMED CT [26]. It could be even possible to compare codes from different terminologies with each other if there is a mapping between them. But this can be a very complex and time-consuming task [30]. Another limitation is that measurement units, which can be stored in ODM for every item, are not analyzed in the current implementation. Thus, it is possible that data for two items, which were found to be identical, cannot be compared. At present, it is difficult to use those measurement units for comparison because they are not fully standardized.

## Supporting Information

S1 File Definition of the compare types. (PDF)

S2 File Tasks for evaluation of ODMSummary v1. (PDF)

S3 File Tasks for evaluation of ODMSummary v2. (PDF)
