# Supplementary material for: ODMSummary: A Tool for Automatic Structured Comparison of Multiple Medical Forms Based on Semantic Annotation with the Unified Medical Language System
Source: PLoS One. 2016 Oct 13;11(10):e0164569. doi: 10.1371/journal.pone.0164569 (PMC5063379; doi:10.1371/journal.pone.0164569)
Supplement: S1 File — (PDF) [file pone.0164569.s001.pdf]

S1 Definition of the compare types

IDENTICAL

- The names of the items are identical (case insensitive).
- The data types of the items are identical.
- The set of UMLS codes assigned to the items is identical.
- If the items refer to a code list:
    - The names of the code lists are identical (case insensitive).
    - The data types of the code lists are identical.
    - The coded values of all code list items are identical (case insensitive).
    - The set of UMLS codes of all code list items is identical.

MATCHING (Data stored for the two items is combinable without transformation.)

- The data types of the items are identical.
- The set of UMLS codes assigned to the items is identical.
- If the items refer to a code list:
    - The data types of the code lists are identical.
    - The coded values of all code list items are identical (case insensitive).
    - The set of UMLS codes of all code list items is identical.

TRANSFORMABLE (Data stored for the two items is combinable using a linear transformation.)

- The set of UMLS codes assigned to the items is identical.
- If the items refer to a code list:
    - The set of UMLS codes of all code list items is identical.

SIMILAR (The concept domain of the two items is identical.)

- The set of UMLS codes assigned to the items is identical.

DIFFERENT (The concept domain of the two items is different.)
