# Supplementary material for: ODMSummary: A Tool for Automatic Structured Comparison of Multiple Medical Forms Based on Semantic Annotation with the Unified Medical Language System
Source: PLoS One. 2016 Oct 13;11(10):e0164569. doi: 10.1371/journal.pone.0164569 (PMC5063379; doi:10.1371/journal.pone.0164569)
Supplement: S2 File — (PDF) [file pone.0164569.s002.pdf]

S2 Tasks for Evaluation of ODMSummary v1

English translation

ODMSummary was developed to compare multiple medical forms in ODM format based on semantic coding with the Unified Medical Language System. Below you will find some tasks to present and evaluate the system. If you would like to participate please fill out the answers to the given tasks or leave comments.

Caution: To fulfill the tasks please do **not** use the Internet Explorer if possible, but Mozilla Firefox or Google Chrome.

Tasks

Task 1: Comparison of multiple versions of one form

Additional to these tasks you got 2 ODM files "Stammdaten und Anamnese v1" and "Stammdaten und Anamnese v5".

1. Please open ODMSummary using the following URL in your browser:
    a. https://odmtoolbox.uni-muenster.de/summary/summary.html
2. Save the 2 ODM files "Stammdaten und Anamnese v1" and "Stammdaten und Anamnese v5" in a folder on your hard drive.
3. Add the 2 files to the comparison:
    a. Click on the file upload field
    b. Choose the folder in which you have stored the 2 files
    c. Select the 2 files (CTRL + mouse click)
    d. Click on „Open"
4. Click the button „Summary".
5. You will see an output with 3 tabs of which you click on the „Compared Items"-tab.
    a. There are the relevant tabs for the tasks
        i. Identical Items,
        ii. Matching Items,
        iii. Transformable Items,
        iv. Similar Items und
        v. Comparable Items
    b. You can check the definition for these comparison types at the end of this document.

Question 1: Which data items were not altered during the development of the form?

Answer:

Task 2: Comparison of forms in the same medical domain

1. Please open the MDM portal in your browser: https://medical-data-models.org
2. Log in (input fields at top of the page).
   a. If you do not have an account you can register by clicking the button „Register“(top of the page). You will get a confirmation mail to verify your email address.
3. If you are logged in the MDM-Portal, click on the “Search“-button.
4. Enter “adt diagnosis“ in the search field “form title“ and click “Search”.
   a. On the right side you will see the result list.
   b. Add the 1st result (quality management ADT-diagnosisdata cancer) to the comparison by clicking on the button (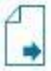) on the right side.
5. Enter “uro cancer conference“ in the search field “form title“ and click “Search”.
   a. On the right side you will see the result list.
   b. Add the 2nd result (HIS cancer conference prostate cancer) to the comparison by clicking on the button (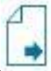 on the right side.
6. Click “Compare Forms“ to perform the comparison.

Question 2: How many reusable (Identical + Matching + Transformable) data items are contained in these 2 forms?

Answer:

Task 3: Comparison of thematically related forms in different languages

1. Please open the MDM portal in your browser: https://medical-data-models.org
2. Log in (input fields at top of the page).
    a. If you do not have an account you can register by clicking the link „Register“ (top of the page). You will get a confirmation mail to verify your email address.
3. If you are logged in in the MDM-Portal, click on the “Search“-button.
4. Enter “cpctr“ in the search field “form title“ and click “Search”.
    a. On the right side you will see the result list.
    b. Add the 1st result (dataset CPCTR CDE Prostata Cancer prostate cancer) to the comparison by clicking on the button (on the right side.
5. Enter “dpkk“ in the search field “form title“ and click “Search”.
    a. On the right side you will see the result list.
    b. Add the 1st result (dataset minimal dataset DPKK prostate cancer) to the comparison by clicking on the button (on the right side.
6. Enter “nct00490139 “ in the search field “form title“ and click “Search”.
    a. On the right side you will see the result list.
    b. Add the 9th result (study documentation NCT00490139 breast cancer) to the comparison by clicking on the button (on the right side.
7. Click “Compare Forms“ to perform the comparison.

Question 3: How many transformable data items are contained in these 3 forms?

Answer:

Task 4: Check forms of a registry for duplicate data collection

1. Please open the MDM portal in your browser: https://medical-data-models.org
2. Log in (input fields at top of the page).
   a. If you do not have an account you can register by clicking the link „Register“(top of the page). You will get a confirmation mail to verify your email address.
3. If you are logged in in the MDM-Portal, click on the “Search“-button.
4. Enter “SHT“ in the search field “form title“ and click “Search”.
   a. On the right side you will see the result list.
   b. Add the complete result list to the comparison by clicking on the buttons (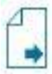) on the right side.
5. Click “Compare Forms“ to perform the comparison.

Question 4.1: Are there data items in the traumatic brain injury registry Münster which are documented twice?

Answer:

Question 4.2 (if applicable): Which data items are documented twice in the traumatic brain injury registry Münster?

Answer:
