# Supplementary material for: ODMSummary: A Tool for Automatic Structured Comparison of Multiple Medical Forms Based on Semantic Annotation with the Unified Medical Language System
Source: PLoS One. 2016 Oct 13;11(10):e0164569. doi: 10.1371/journal.pone.0164569 (PMC5063379; doi:10.1371/journal.pone.0164569)
Supplement: S3 File — (PDF) [file pone.0164569.s003.pdf]

S3 Tasks for Evaluation of ODMSummary v2

English translation

ODMSummary was developed to compare multiple medical forms in ODM format based on semantic coding with the Unified Medical Language System. Below you will find some tasks to present and evaluate the system. If you would like to participate please fill out the answers to the given tasks or leave comments. Also you can comment the given Excel files concerning data you do not understand. Please send all files even if they were not commented.

Tasks

Task 1: Comparison of multiple versions of one form

1. Please open the file "Aufgabe1_IdenticalItems.xlsx".
2. Use the output in this file to answer the following question, and
3. mark all fields in the output you do not understand and leave a comment.

Question 1: Which data items were not altered during the development of the form?

Answer:

Task 2: Comparison of forms in the same medical domain

1. Please open the files
    a. "Aufgabe2_IdenticalItems.xlsx" and
    b. "Aufgabe2_MatchingItems.xlsx".
    Hint: Transformable data items are not contained in these forms.
2. Use the output in this file to answer the following question, and
3. mark all fields in the output you do not understand and leave a comment.

Question 2: How many reusable (Identical + Matching + Transformable) data items are contained in these 2 forms?

Answer:

Task 3: Comparison of thematically related forms in different languages

1. Please open the file "Aufgabe3_TransformableItems.xlsx".
2. Use the output in this file to answer the following question, and
3. mark all fields in the output you do not understand and leave a comment.

Question 3: How many transformable data items are contained in these 3 forms?

Answer:

Task 4: Check forms of a registry for duplicate data collection

1. Please open the file “Aufgabe4_IdenticalItems.xlsx”.
2. Use the output in this file to answer the following questions, and
3. mark all fields in the output you do not understand and leave a comment.

Question 4.1: Are there data items in the traumatic brain injury registry Münster which are documented twice?

Answer:

Question 4.2 (if applicable): Which data items are documented twice in the traumatic brain injury registry Münster?

Answer:
